# Does Combining the STarT Back Tool With a Polygenic Risk Score for Chronic Low Back Pain Improve Prediction of Work Disability Over 2 Years?

**DOI:** 10.1002/ejp.70257

**Published:** 2026-03-26

**Authors:** Roger Compte, Maryam Kazemi Naeini, Eveliina Heikkala, Terence McSweeney, Jaro Karppinen, Frances M. K. Williams

**Affiliations:** ^1^ Department of Twin Research and Genetic Epidemiology, School of Life Course and Population Sciences King's College London London UK; ^2^ Research Unit of Health Sciences and Technology University of Oulu Oulu Finland

## Abstract

**Introduction:**

Chronic back pain (CBP) is a leading cause of work disability worldwide, yet identifying individuals at risk remains difficult due to its multifactorial aetiology. This population‐based cohort study investigated whether integrating a polygenic risk score (PRS) for CBP with the STarT Back Tool (SBT)—a widely used psychosocial screening instrument—could improve the prediction of work disability, measured as disability leave days over a 2‐year follow‐up.

**Methods:**

We analysed data from 1938 participants in the Northern Finland Birth Cohort 1966 with complete genotyping, SBT responses and registry‐linked disability records. A zero‐inflated negative binomial regression model was applied to account for the highly skewed distribution of work disability days.

**Results:**

Results showed that both SBT and CBP genetic risk independently predicted the cumulative number of disability leave days. While SBT was also associated with the likelihood of having no disability leave, CBP genetic risk was not, suggesting that polygenic risk contributes specifically to the burden of disability among affected individuals. When participants split into 4 risk groups, those in the highest CBP genetic risk quartile experienced significantly more work disability days.

**Conclusion:**

The two tools captured complementary domains: SBT reflected modifiable biopsychosocial risks, while the PRS represented fixed genetic liability. This distinction supports the value of integrating a CBP PRS into existing screening frameworks, with potential in early CBP management.

**Significance Statement:**

This study is the first to combine a polygenic risk score for chronic back pain with a clinical screening tool to assess work disability outcomes. It demonstrates that genetic and psychosocial risk capture distinct aspects of vulnerability, and that their integration improves risk stratification. These findings add to the growing evidence supporting personalized approaches in pain management and highlight the potential utility of genetic data in early assessment of disabling back pain.

## 1 Introduction

Chronic low back pain (CBP) remains a clinical and socioeconomic challenge, ranking as the leading cause of disability worldwide (Ferreira et al. 2023). Musculoskeletal disorders, including CBP, are the main reason for early exclusion from the labour market due to permanent disability (Finnish Centre for Pensions 2024). Beyond structural factors like spine pathologies and degenerative conditions, other aspects such as biomechanical stresses, psychological states and genetic predisposition further complicate CBP (Freidin et al. 2019; Tegeder and Lötsch 2009). Psychological factors, including stress and anxiety, amplify pain perception and contribute to pain chronicity (Pincus et al. 2002). Depression, metabolic conditions, such as high body mass index (BMI) and diabetes, and smoking are among other important risk factors for CBP (Nieminen et al. 2021). These and other biopsychosocial variables have been integrated into validated models that reliably estimate the risk and progression of chronic pain (Tanguay‐Sabourin et al. 2023).

A key challenge in managing low back pain lies in understanding the transition from acute to chronic stages. Although CBP is traditionally defined by its persistence beyond 3 months, this transition reflects more than just temporal duration—it involves a complex interplay of ongoing nociceptive input, central nervous system plasticity, psychological factors and individual biological vulnerabilities, including genetic, endocrine and immune system influences (Zimney et al. 2023). Notably, the heritability of CBP‐related disability has been estimated at approximately 40% (Battié et al. 2007). Genome‐wide association studies (GWAS) have been instrumental in elucidating the genetic architecture of complex diseases like CBP by identifying genetic variants associated with disease susceptibility. These studies suggest that CBP genetic factors may influence not only pain perception but also broader domains such as psychiatric, sociodemographic and anthropometric traits, including depression, educational attainment and BMI (Freidin et al. 2019; Stanaway et al. 2025).

In primary care settings, the STarT Back Tool (SBT) is widely utilised to stratify acute low back pain patients according to their risk of chronicity (Hill et al. 2008). SBT integrates physical symptoms (pain intensity, functional impairment) and psychosocial factors (anxiety, depression, fear‐avoidance beliefs) to categorise patients into low‐, medium‐ and high‐risk groups. This risk stratification aims to guide treatment intensity—from general advice for low‐risk patients to psychologically informed physiotherapy for high‐risk patients—to prevent the development of CBP (Hill et al. 2008). Despite its international use, evidence for the SBT's predictive performance is mixed: it predicts disability reasonably well in primary care and disability (Karran et al. 2017; Kendell et al. 2018) but performs less well for pain (Karran et al. 2017) and work‐related (Unsgaard‐Tøndel et al. 2021) outcomes in other settings and populations (Morsø et al. 2014; Vigdal et al. 2024).

Polygenic risk scores (PRS), developed from GWAS, aggregate genetic risk variants to estimate individual disease risk. PRS are used for patient stratification, to enhance diagnostic accuracy and to predict outcomes from interventions or assist in subsequent monitoring (Zheutlin and Ross 2018). For example, in cardiovascular medicine, integrating ancestry‐specific PRS into existing clinical risk models improved prediction of coronary artery disease, particularly in individuals at borderline and intermediate clinical risk—precisely where decision‐making is often most uncertain (Ratman et al. 2025).

The primary objective of this population‐based cohort study was to investigate whether combining a CBP polygenic risk score with SBT outcomes could improve risk stratification for work disability, specifically measured as disability leave days over a 2‐year follow‐up period using national register data. We hypothesised that integrating genetic risk information would provide complementary insights to the physical and psychosocial dimensions currently assessed in clinical evaluation.

## 2 Methods

### 2.1 Study Sample

The study sample was drawn from the Northern Finland Birth Cohort 1966 (NFBC1966) (Nordström et al. 2022; University of Oulu 2024), which consists of individuals born in two provinces of Finland, Oulu and Lapland, whose mothers had expected delivery dates in 1966. The cohort initially included 12,068 pregnant women, resulting in 12,231 children, covering 96.3% of all births in the region that year. Since their mothers' first antenatal clinic visit, repeated data collections have tracked cohort members longitudinally.

During the 46‐year follow‐up phase from 2012 to 2014, when the participants were 45–47 years old, those still alive and with known contact details were invited to participate in a health survey, either electronically or by post, and attend a clinical examination. A total of 7148 participants responded to the questionnaire; of those, 3403 reported having LBP within the past 12 months (47.6%) and responded to the SBT questionnaire. To contextualise this prevalence estimate, a comprehensive systematic review by Hoy et al. (2012) reported a global 1‐year prevalence of 38% (SD ±19.4) for any back pain. For low back pain specifically, the review estimated a pooled mean prevalence of 29.1% (SD ±18.8) across studies. The NFBC 1‐year prevalence falls within the broad range reported in international studies, although near the upper end. This is consistent with the use of self‐reported questionnaires and with the cohort's midlife age, both factors known to increase back pain reporting. Data from the questionnaire and clinical assessments were then linked to register‐based records on sick leave and disability pensions for those who provided written consent.

**FIGURE 1:**
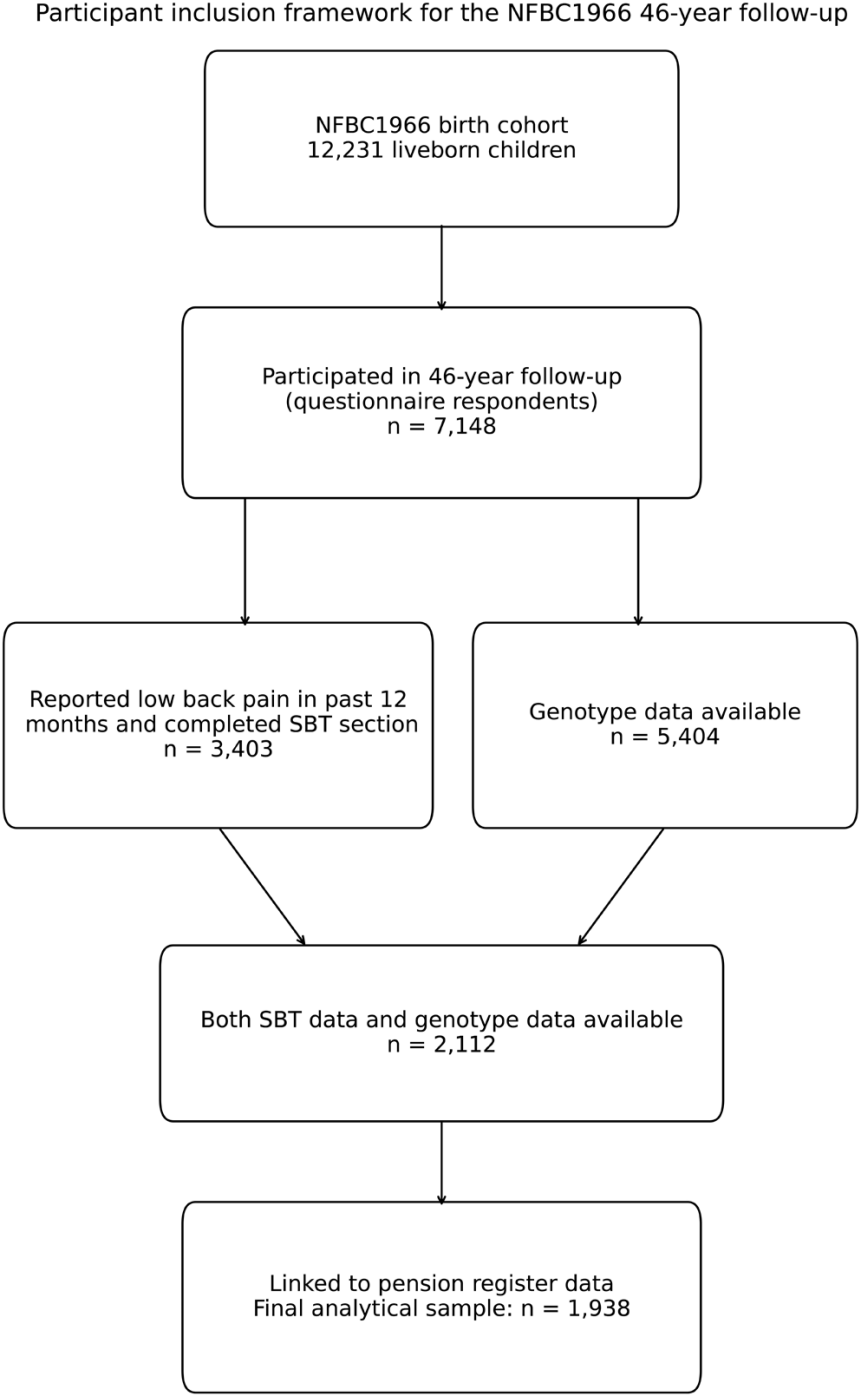
Flow diagram showing derivation of the final analytical sample at the 46‐year follow‐up of the NFBC1966 cohort. The final sample included 1938 participants with SBT data, genotype data and successful linkage to pension registers.

A total of 5404 participants from NFBC1966 were genotyped using Illumina Human CNV370‐Duo DNA bead chip as previously described elsewhere (Sabatti et al. 2009) and imputed using IMPUT2 and 1000 Genomes phase 3 reference panel. Overall, 2112 had both genotype data and responded to the SBT questionnaire. From those, 1938 participants were successfully linked to sick leave and disability pension register data and were not on a disability pension at baseline (Figure 1). Each participant was individually tracked from the registers over a 2‐year follow‐up period starting the day after completing the baseline questionnaire. If this was not known (a participant had not reported it), the clinical examination date was used.

The study followed the principles of the Declaration of Helsinki. The participants took part on a voluntary basis and signed informed consent forms. The Northern Ostrobothnia Hospital District Ethical Committee has approved the study, 94/2011 (12.12.2011).

### 2.2 Work Disability Days

The assessment of disability‐related days absent from work involved the aggregation of all sickness absence days and disability pension days into a single ‘work disability days’ variable, as used in a prior NFBC1966 study by Varanka‐Ruuska et al. (2020). Data for this variable were extracted from two national Finnish registers, the Finnish Centre for Pensions (Finnish Centre for Pensions 2024) (FCP) and the Social Insurance Institution of Finland (SII, Kela register) (The Social Insurance Institution of Finland 2024).

To accurately capture the data, it was noted that in Finland, sick leave days are registered into the FCP and Kela registers only after varying deductible times. For the Kela register, only sick leave periods exceeding 10 days (or 4 days for entrepreneurs) are recorded, while for the FCP register, which primarily includes data on sick leave due to accidents, the first 4 days are covered by employees and are not recorded. Both full‐ and part‐time sick leave days were considered to be included in the sick leave days outcome. Participants were considered to have a disability pension day if they were granted either a fixed‐term or permanent disability pension, regardless of whether it was full‐time or part‐time. In most cases, to be able to get disability pension benefits in Finland, a person must have been on a sick leave for 1 year before the eligibility for disability pension is evaluated.

### 2.3 Polygenic Risk Score (PRS)

Tsepilov et al. (2023) recently developed the PRS specifically for CBP using GWAS data from the UK Biobank cohort. Briefly, the PRS was derived from a CBP GWAS that used genotype data imputed via IMPUT2 software and the 1000 Genomes phase 3 reference panel from the UK Biobank cohort. Consistent with prior methods, PRS values were standardised using a *z*‐score transformation to facilitate comparison across participants. While the CBP PRS demonstrated moderate predictive ability (AUC = 0.56; 95% CI: 0.56–0.57), its validation across independent cohorts reinforces its potential for clinical application.

### 2.4 STarT Back Tool

Participants completed a validated Finnish version of the SBT (Piironen et al. 2016). The original version is provided in Methods S1 and the translated version is provided in methods S2. SBT scores were calculated by summing positive responses to the nine questionnaire items, resulting in total scores ranging from 0 to 9, with higher scores indicating increased risk of chronic back pain. The nine questions evaluate leg pain referral, comorbid pain in the neck or shoulder, disability in daily activities, pain bothersomeness and five psychosocial domains: fear, anxiety, catastrophising, depression and low confidence in activity.

### 2.5 Covariates

Sex was recorded at birth, thus dichotomised as female and male. BMI was calculated using weight and height measured at clinical examination. Participants were grouped as ‘non‐smokers’, ‘former smokers’ or ‘current smokers’ based on how they had responded to the questions: ‘Have you ever been a regular smoker?’ and ‘Do you smoke now?’ Educational attainment was classified into two levels based on total years of schooling completed by age 46: 12 years or less, including no formal education beyond secondary education; and 13 or more years, representing post‐secondary or higher education. Occupational status was obtained from the questionnaire and classified into unemployed, employed (working full‐time or part‐time, self‐employed or entrepreneur, employed/educated by labour market support) and other status such as students, on parental/sabbatical leave, homemakers or other activities not listed before. In Finland, disability pension is possible irrespective of current employment status; therefore, participants classified as unemployed were not automatically assigned zero disability‐days and could contribute non‐zero pension days in our outcome variable.

All variables included in the regression analyses had complete data after restricting to participants with full covariate information.

### 2.6 Association Analysis

Initial exploratory and crude associations among variables were assessed using Spearman's correlation. Work disability days presented a skewed distribution and high frequency of zeros. To address potential biases from excess zero counts, we applied a zero‐inflated negative binomial (ZINB) regression model, first in a univariate exploratory analysis and then in the multivariate main analysis. This enabled separate modelling of (a) factors associated with the occurrence (logistic component) and (b) factors predicting the total day number (negative binomial component) of work disability.

Exponentiated coefficients from the negative binomial component from the ZINB model are presented as rate ratios (RRs). The outcome modelled, total work disability days, is technically a duration measure rather than a discrete event count, the negative binomial framework models it as count data for statistical purposes. Using the term ‘incidence rate ratio’ (IRR) would be misleading in this context, as our outcome does not reflect new event occurrences over person‐time. Therefore, we refer to the exponentiated estimates as ‘rate ratios’ (RRs) to more accurately describe the multiplicative change in expected work disability days associated with a one‐unit change in each predictor variable. For the logistic component of the zero‐inflated model, results are presented as odds ratios (ORs), representing the odds of having no disability leave days (i.e., belonging to the structural zero group) associated with each predictor. An odds ratio greater than 1 indicates higher odds of no disability leave, while an odds ratio less than 1 indicates lower odds. Confidence intervals (95% CIs) for both RRs and ORs were calculated using the standard normal approximation, based on ±1.96 times the standard error of the coefficient estimates.

Zero‐inflated regression analyses were performed using the zeroinfl function from the pscl package in R, specifying negative binomial distribution for the count component. Covariates included in all multivariate regression analyses were sex, BMI, smoking status (never, former or current smoker), education level (up to secondary education or post‐secondary education) and occupational status (employed, unemployed or other). Smoking status, occupational status, sex and educational level were considered categorical variables; the remaining variables including work disability days, SBT, PRS and BMI were treated as continuous variables.

To explore possible non‐linear effects, participants were categorised into PRS quartiles, allowing investigation of disability leave days across different levels of genetic risk. All analyses were performed using *R* statistical software. Statistical significance was defined at the conventional threshold of *p* < 0.05. Univariate analyses were used to describe crude associations; the multivariate model represented the primary adjusted analysis, and the categorised PRS model was included to visualise the pattern of association rather than to perform an additional significance test.

To visualise the effect of SBT and PRS risk groups on the total number of work disability days, the expected work disability days were calculated at the different values of those variables, keeping all other confounders constant and using the negative binomial estimates of the ZINB. In particular, expected work disability days at different SBT and PRS risk groups were calculated for an employed non‐smoker male with the sample mean BMI and no higher education. Predicted values were obtained by setting the covariates to these reference levels and varying SBT and PRS categories accordingly, using the fitted model to compute marginal means.

This study follows the STROBE reporting guideline for observational research. A completed STROBE checklist is provided in Methods S3.

## 3 Results

### 3.1 Study Sample and Main Data

Among the 1938 NFBC1966 subjects with available SBT, PRS and leave days data, 1713 had complete information on sex, BMI, smoking status, education level and occupational status. The descriptors are summarised in Table 1.

**TABLE 1:** Table presenting the main descriptors of the sample characteristics including main confounder variables.

Category	Statistics
Sample (complete data)	1713 subjects, 46 years old, 56.3% female
Work disability days (all subjects)	0–730 days; 75.6% had no disability leave days
Work disability days (with disability)	Median 67.95 days (IQR 61.50)
SBT score	0–9 range; mean 1.6 ± 1.6
Chronic back pain PRS	−3.32 to 3.47 (*z*‐score normalised)
BMI (mean ± SD)	26.8 ± 4.8
Smoking status	47% never, 30% former, 23% current smokers
Education (post‐secondary)	25%
Employment	91% employed, 4% unemployed, 5% other status

Mean age was approximately 46 years at the time of sampling. The sample was 56.3% female, with a mean BMI of 26.8 kg/m^2^ (SD = 4.8). Among participants with complete data, 47% had never smoked and 23% were current smokers. Seventy‐five percent of the sample had up to secondary education, and only 4% of the participants were unemployed at the time of the questionnaire. Work disability ranged from 0 to 730 days, with 75.6% presenting no work disability accumulated over 2‐year period. Subjects with work disability had a median of 67.95 days and an inter‐quartile range of 61.50 days. SBT questionnaire responses were summed to yield a score ranging from 0 to 9, with a mean of 1.6 (SD = 1.6). The CBP PRS was normally distributed and normalised using a *z*‐score, ranging from −3.32 to 3.47. Distributions of disability leave days, SBT summed scores and PRS scores are shown in Figure S1.

### 3.2 Univariate Analysis

**FIGURE 2:**
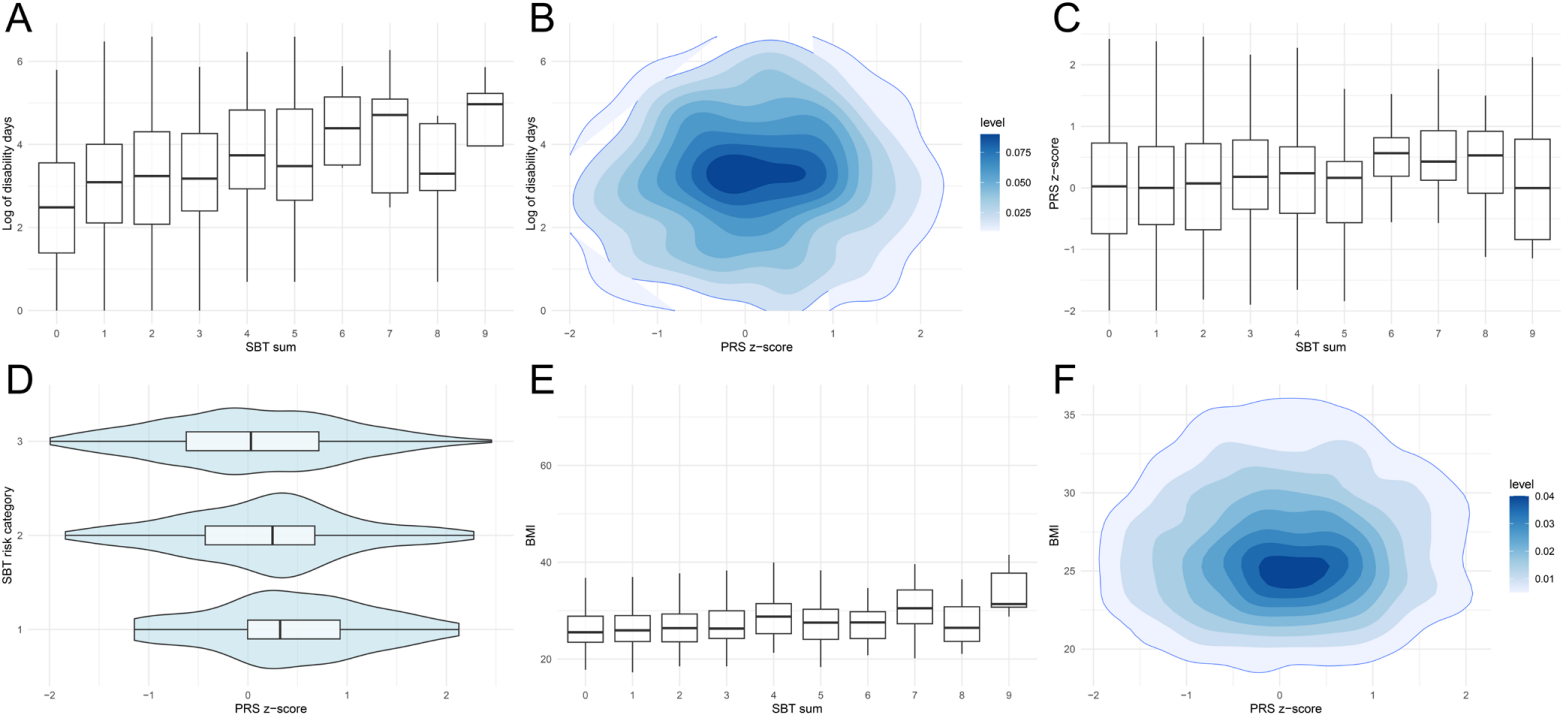
Pairwise correlations among variables. Distributions of disability leave days by SBT scores and PRS (A, B); distribution of PRS by SBT scores and SBT‐defined chronicity risk groups (C, D); distribution of BMI by SBT scores and PRS (E, F).

In the univariate analysis, higher SBT scores were correlated with greater work disability days (Spearman's correlation coefficient ρ = 0.185, *p* = 2.14 × 10^−17^; Figure 2A). In univariate ZINB regression, increased SBT scores were associated with a higher total number of work disability days and a lower likelihood of having no disability leave (RR = 1.17, 95% CI: 1.08–1.28; OR = 0.78, 95% CI: 0.73–0.85). In contrast, CBP PRS was not significantly correlated with work disability days (Spearman's ρ = 0.015, *p* = 0.54; Figure 2B). Regression analysis showed that genetic risk was not associated with the likelihood of having zero work disability days (OR = 0.98, 95% CI: 0.87–1.11), although among individuals with disability, higher genetic risk was associated with a greater total number of work disability days (RR = 1.35, 95% CI: 1.15–1.60). Additionally, CBP PRS was weakly but significantly correlated with higher SBT scores (ρ = 0.08, *p* = 0.002; Figure 2C) and with higher SBT‐predicted risk group classifications (low, medium and high risk; ρ = 0.070, *p* = 0.004; Figure 2D).

Higher SBT scores were also correlated with increased BMI (ρ = 0.12, *p* = 5.847 × 10^−7^; Figure 2E), while no significant correlation was found between PRS and BMI (*ρ* = 0.03, *p* = 0.16; Figure 2F). SBT was further correlated with lower education level (*ρ* = −0.16, *p* < 0.001), unemployed occupational status (*ρ* = −0.10, *p* < 0.001), female sex (ρ = 0.067, *p* = 0.006) and smoking status (*ρ* = 0.09, *p* = 1.81 × 10^−4^). In contrast, the PRS was not significantly correlated with sex (*ρ* = 0.023, *p* = 0.35) or education level (*ρ* = −0.012, *p* = 0.61), but showed weak correlations with unemployed occupational status (*ρ* = −0.052, *p* = 0.033) and smoking status (*ρ* = 0.07, *p* = 0.008).

To evaluate model fit, we compared the SBT‐only model to a model including both SBT and PRS using average AIC values from 5‐fold resampling (ZINB model). The addition of PRS reduced the average AIC from 4843.37 to 4833.54, indicating a modest improvement in model fit.

### 3.3 Multivariate Analysis

In ZINB multivariate analysis, SBT was a significant predictor in both components of the model similarly to univariate analysis. Higher SBT scores were associated with a longer cumulative duration of work disability (RR = 1.16, 95% CI: 1.08–1.26) and with a lower likelihood of having zero disability days (OR = 0.81, 95% CI: 0.75–0.87). Likewise, higher PRS scores were associated with an increased total number of work disability days (RR = 1.33, 95% CI: 1.17–1.51) but not with changes in the likelihood of no disability. Never smokers and employed individuals were associated with a lower number of work disability days compared to current smokers and unemployed, respectively. Higher BMI showed a small but statistically significant association with a lower likelihood of having zero work disability days (OR = 0.96, 95% CI: 0.94–0.99), but among individuals who took work disability days, it was also associated with a modest reduction in the total number of work disability days (RR = 0.96, 95% CI: 0.94–0.98). Additionally, males were more likely to have no recorded work disability than females. The effect estimates and statistical significance of all predictors from the adjusted zero‐inflated regression are summarised in Table 2.

**TABLE 2:** Statistics from the zero‐inflated regression of work disability days using SBT, PRS as predictors including adjustments for sex, smoking status, employment status and BMI.

Negative binomial regression predicting total work disability days (count distribution)				
	RR	95% CI		*p*
SBT score	1.16	1.08	1.26	0.000
PRS	1.33	1.17	1.51	0.000
Sex (male)	0.80	0.60	1.06	0.124
Ex‐smoker (vs. current smoker)	1.14	0.80	1.64	0.458
Never smoked (vs. current smoker)	0.61	0.43	0.85	0.004
Post‐secondary education	1.34	0.95	1.89	0.092
Employed (vs. unemployed)	0.32	0.16	0.65	0.002
Other employed status (vs. unemployed)	0.82	0.45	1.49	0.521
BMI	0.96	0.94	0.99	0.016
Dispersion parameter estimate (*θ*)	0.49	0.40	0.60	0.000

Logistic regression predicting probability of no disability days (structural zeros)				
	OR	95% CI		*p*
SBT score	0.81	0.75	0.87	0.000
PRS	1.04	0.92	1.17	0.544
Sex (male)	1.40	1.10	1.79	0.007
Ex‐smoker (vs. current smoker)	1.03	0.74	1.42	0.840
Never smoked (vs. current smoker)	1.28	0.95	1.74	0.109
Post‐secondary education	1.29	0.96	1.73	0.086
Employed (vs. unemployed)	0.71	0.38	1.33	0.282
Other employed status (vs. unemployed)	1.23	0.53	2.88	0.540
BMI	0.96	0.94	0.98	0.001

Abbreviations: BMI, body mass index; CI, confidence intervals; OR, odds ratio; PRS, polygenic risk score; RR, rate ratio; SBT, STarT Back Tool.

**FIGURE 3:**
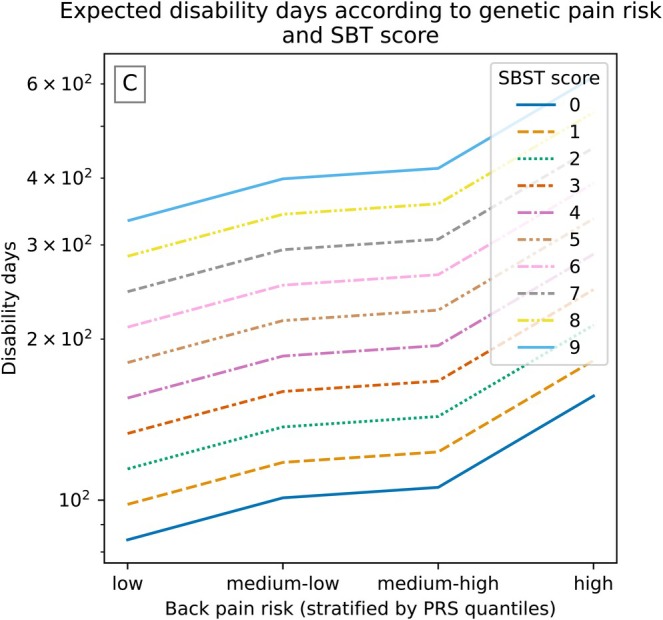
Predicted work disability days based on regression estimates. Expected disability days (log scale, *y*‐axis) across genetic risk groups (*x*‐axis, stratified by PRS quantiles), with lines representing SBST scores from 0 to 9. Uncertainty in prediction from parameter variance is omitted to maintain visual clarity and emphasise the functional shape of the modelled relationship.

To aid interpretation and explore potential non‐linearity in the association, the PRS was additionally categorised into four groups based on sample quartiles. These PRS groups were incorporated into the multivariate model in place of the continuous score. The overall pattern was consistent with the main analysis: individuals in the highest PRS group had significantly more work disability days than those in the lowest group (RR = 1.86, 95% CI: 1.25–2.76), whereas the two intermediate groups did not differ statistically from the reference. Nevertheless, the predicted mean disability days increased progressively across the PRS quartilesThe SBT associations remained unchanged (RR = 1.16, 95% CI: 1.07–1.27; OR = 0.81, 95% CI: 0.75–0.87; see Table S1 for all variables). Figure 3 illustrates the predicted mean work disability days across PRS groups and SBT scores.

## 4 Discussion

CBP remains a significant clinical and socioeconomic burden, underscoring the need for accurate early risk stratification tools to guide management and reduce disability. This study demonstrates that integrating a PRS for CBP with the established SBT adds to the characterisation of work disability outcomes, specifically measured as the number of work disability days over a 2‐year period.

The PRS was a significant predictor of total work disability days in both univariate and multivariate zero‐inflated models (RR = 1.35, 95% CI: 1.15–1.60; RR = 1.33, 95% CI: 1.17–1.51, respectively), despite showing no significant overall association with the occurrence of work disability. When examined in quartiles, the predicted disability days increased numerically across PRS groups, although only the comparison between the highest and lowest quartiles reached statistical significance. This pattern is compatible with a small underlying gradient in genetic risk, where differences across intermediate levels may be too modest to detect individually but become apparent when contrasting the extremes of the PRS distribution. Such a pattern may also reflect a non‐linear or threshold‐type effect, with greater disability burden emerging primarily among individuals with the highest levels of genetic liability. In contrast, the SBT score was overall significantly correlated with work disability days (Spearman's coefficient = 0.185, *p* = 2.14 × 10^−17^), and consistently associated with disability outcomes in both univariate and multivariate analyses. Higher SBT scores were robustly linked to higher number of work disability days (RR = 1.17, 95% CI: 1.08–1.28; RR = 1.16, 95% CI: 1.08–1.26, respectively) and with a reduced likelihood of having zero work disability days(OR = 0.78, 95% CI: 0.73–0.85; OR = 0.81, 95% CI: 0.75–0.87, respectively). Importantly, the improvement in prediction model fit with the addition of PRS was modest, indicating that genetic risk provides an incremental contribution rather than a stand‐alone or practice‐changing predictor at this stage. Our use of zero‐inflated negative binomial regression allowed us to disentangle predictors of the likelihood of experiencing any work disability (logistic component) from those affecting its total cumulative duration (count component). Notably, genetic risk was only associated with the severity (i.e., number of work disability days) of disability, not the probability of having no disability days. This distinction suggests that genetic predisposition may play a larger role in determining the chronicity of back pain rather than the initial occurrence of disability. Notably, the logistic component does not strictly model disability initial occurrence but no‐disability occurrence, and such interpretation should be considered inferential. Conversely, SBT captures both functional and psychosocial aspects that influence both risk and persistence of disability. Together, these patterns support the view that PRS and SBT tap into distinct but complementary domains of vulnerability such as biological versus biopsychosocial respectively.

This interpretation is further reinforced by the finding that SBT scores were significantly correlated with BMI—a well‐established metabolic and physical risk factor for disability—whereas PRS showed no such relationship. More broadly, SBT demonstrated consistent correlations with several modifiable sociodemographic and behavioural factors, including education, occupational status and smoking, as well as non‐modifiable traits like sex. In contrast, PRS was largely uncorrelated with these variables, showing only weak associations with occupational status and smoking. Nevertheless, the correlation of back pain genetic risk with smoking is not unexpected, as genetic correlation between back pain and smoking has already been reported (Freidin et al. 2019). These differences highlight how the two measures complement each other: SBT reflects mainly changeable, real‐world factors influencing disability—and probably pain—while PRS captures mostly fixed genetic risk.

When PRS was analysed in quartiles, individuals in the highest genetic risk group showed clearly higher work disability days compared with those in the lowest group, underscoring the relevance of genetic risk in understanding the burden of disability. While PRS was not associated with the likelihood of having zero disability days, it effectively predicted the burden among those who were affected—a pattern consistent with SBT's role in early care settings, where symptoms have already emerged. Importantly, these findings highlight the potential for PRS to enrich existing screening approaches at population level. Adding genetic data to clinical tools like SBT could help identify people at risk of prolonged work disability. Naturally, further studies in patient samples are needed but this integration may have the potential to support more personalised treatment planning, including earlier referrals for intensive interventions among those genetically predisposed to worse outcomes.

In addition to clinical tools and genetic predisposition, our study identified modifiable predictors associated with work disability outcomes. Never smoking and being employed were linked to a lower number of work disability days, emphasising the protective effects of healthier lifestyle behaviours. Although higher BMI was associated with a lower likelihood of having zero disability days, it was also linked to a modest reduction in the total duration of the work disability among those affected. This nuanced finding suggests that while BMI may influence the risk of disability occurrence, it has a more complex or less straightforward relationship with the severity of work disability once it occurs. One speculative explanation is that occupational health practices in Finland, such as the implementation of alternative duty work policies, may be more frequently applied to individuals with obesity, given that obesity is a well‐recognised and visible risk factor for reduced work ability. These policies allow employees to perform modified duties instead of taking sick leave, which may contribute to a shorter duration of work disability in this group. Additionally, males showed a higher probability of not registering disability leave compared to females. These findings highlight the important role of modifiable social and behavioural factors in shaping disability outcomes and suggest potential targets for intervention alongside clinical and genetic risk stratification.

The strengths of our study include the use of objectively recorded disability data from national registries, a large and representative population‐based cohort, and the application of advanced modelling techniques that account for the zero‐inflated nature of work disability days data. To our knowledge, this is the first study to empirically test and demonstrate the value of integrating a validated PRS for back pain with the SBT tool, offering early evidence that such integration may meaningfully improve risk stratification in primary care. Although the cohort's 1‐year prevalence of low back pain may appear high, this does not affect our findings. Our analyses were intentionally restricted to individuals reporting recent low back pain—the appropriate target population for applying the SBT—and therefore prevalence estimates at the full‐cohort level are not directly meaningful. If anything, including participants without low back pain would likely increase the proportion of low‐risk SBT profiles and broaden generalisability.

Nonetheless, certain limitations must be acknowledged. First, the outcome measure—total work disability days—did not distinguish between causes of disability, meaning that not all disability days may have been directly attributable to back pain. Unfortunately, diagnosis‐specific sick leave information (e.g., musculoskeletal vs. non‐musculoskeletal causes) was not available in the registers used, and therefore sensitivity analyses restricted to musculoskeletal causes could not be performed. This lack of specificity may have introduced some misclassification, as a proportion of recorded disability leave may reflect other health conditions. However, because this imprecision likely applies similarly across all participants—regardless of their SBT score or genetic risk—any bias would tend to weaken, rather than inflate, the observed associations. In other words, the true effects of these predictors on back pain–related disability may be stronger than what we report. Future studies incorporating diagnostic codes (e.g., ICD‐10 or ICD‐11) could offer greater specificity, but given that chronic back pain is a leading cause of work disability, our broader measure remains clinically relevant and justifiable for this setting. Second, the observational nature of the study limits causal inference. Although we adjusted for major confounders, the possibility of residual confounding or unmeasured variables cannot be excluded. Finally, although the SBT captures biopsychosocial factors, it does not explicitly include occupational or work‐related determinants, which may also influence disability outcomes (Unsgaard‐Tøndel et al. 2021).

In conclusion, this study provides novel evidence that genetic risk information captures an additional dimension of vulnerability to work disability beyond established clinical screening. While the incremental contribution of genetic risk is modest, it highlights a complementary biological dimension not captured by clinical tools alone and further research is required to refine prediction models and validate these findings across broader populations and outcome measures. These findings support further investigation into how genetic information may refine risk stratification and inform future, more personalised approaches to managing back pain–related disability.

## Author Contributions

This study was designed by R.C., M.K.N. and F.M.K.W. The data were analysed by R.C., M.K.N., E.H. and T.M. The results were critically examined by all authors. R.C. had a primary role in preparing the manuscript, which was reviewed and edited by J.K. and F.M.K.W. All authors have approved the final version of the manuscript and agree to be accountable for all aspects of the work.

## Funding

This project was supported by the Marie Skłodowska Curie International Training Network (ITN) ‘disc4all’ (https://disc4all.upf.edu, accessed on 1st April 2025) grant agreement #955735. NFBC1966 46y follow‐up study received financial support from University of Oulu Grant no. 24000692, Oulu University Hospital Grant no. 24301140, ERDF European Regional Development Fund Grant no. 539/2010 A31592.

## Conflicts of Interest

The authors declare no conflicts of interest.

## Supporting information


**Figure S1:** Distributions of the main variables.
**Figure S2:** Predicted work disability days based on regression estimates.
**Table S1:** Statistic of the regression of disability days using stratified PRS by quantiles in four risk groups.
**Methods S1:** The original English version of the STarT Back Tool (SBT) questionnaire.
**Methods S2:** Finnish version of SBT.

## Data Availability

NFBC data are available from the University of Oulu, Infrastructure for Population Studies. Permission to use the data can be applied for research purposes via an electronic material request portal. In the use of data, we follow the EU general data protection regulation (679/2016) and the Finnish Data Protection Act. The use of personal data is based on a cohort participant's written informed consent in their latest follow‐up study, which may cause limitations to its use. Please contact the NFBC project center (nfbcprojectcenter@oulu.fi) and visit the cohort website (www.oulu.fi/nfbc) for more information.
