# Supplementary material for: Does Combining the STarT Back Tool With a Polygenic Risk Score for Chronic Low Back Pain Improve Prediction of Work Disability Over 2 Years?
Source: Eur J Pain. 2026 Mar 26;30(4):e70257. doi: 10.1002/ejp.70257 (PMC13019420; doi:10.1002/ejp.70257)
Supplement: Supplementary file 1 — Figure S1: Distributions of the main variables. Figure S2: Predicted work disability days based on regression estimates. Table S1: Statistic of the regression of disability days using stratified PRS by quantiles in four risk groups. Methods S1: The original English version of the STarT Back Tool (SBT) questionnaire. Methods S2: Finnish version of SBT. [file EJP-30-0-s001.pdf]

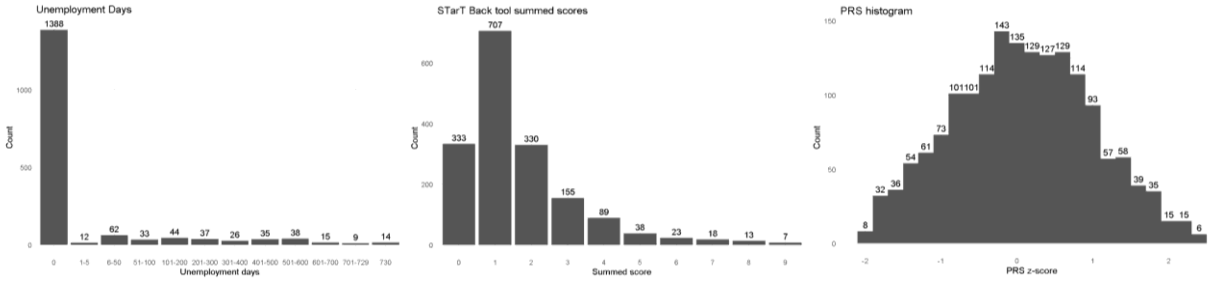

Figure S1. Distributions of the main variables. Disability leave days (left); STarT Back Tool summed score (SBT, middle); chronic back pain polygenic risk score (PRS, right).

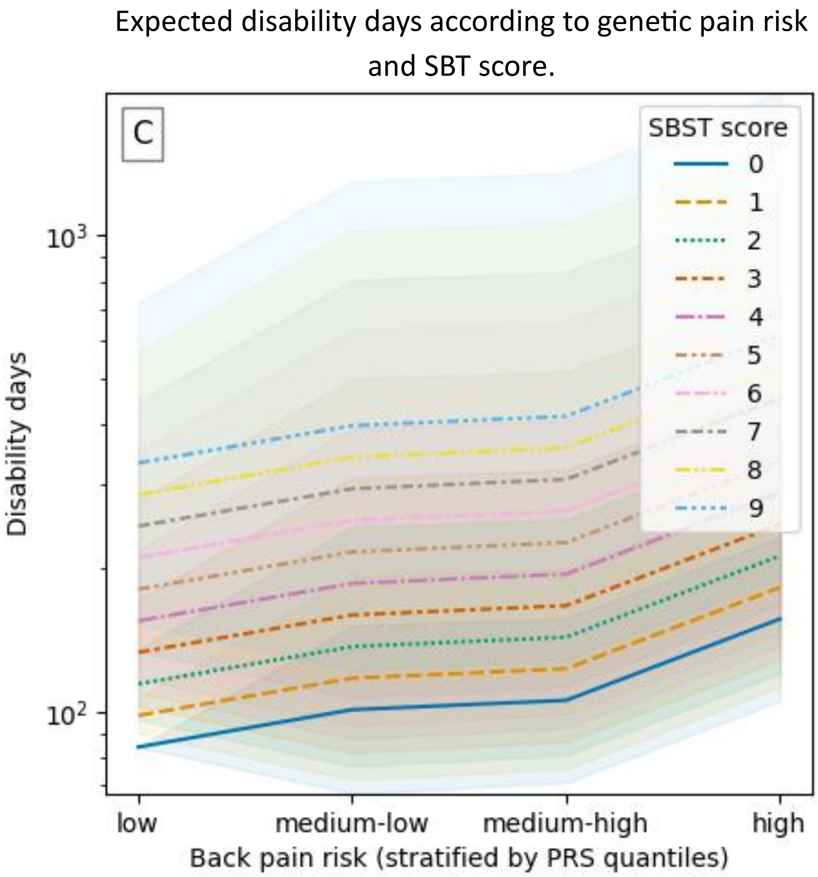

Figure S2. Predicted work disability days based on regression estimates. Expected disability days (log scale, y-axis) across genetic risk groups (x-axis, stratified by PRS quantiles), with lines representing SBST scores from 0 to 9. Shaded areas correspond to 95% confidence intervals, calculated by predicting disability days using the lower and upper bounds of the parameter confidence intervals.

Table S1. Statistic of the regression of disability days using stratified PRS by quantiles in four risk groups. BMI: body mass index; PRS: polygenic risk score, SBT: STarT Back Tool; RR: rate ratio; OR: odds ratio; CI: confidence intervals.

Negative binomial regression predicting total disability leave days (count distribution)

	RR	95% CI		p-value
**SBT score**	1.16	1.07	1.27	0.001
**PRS risk medium-low (vs low)**	1.20	0.80	1.80	0.384
**PRS risk medium-high (vs low)**	1.25	0.84	1.86	0.301
**PRS risk high (vs low)**	1.86	1.25	2.76	0.002
**Sex (male)**	0.83	0.62	1.11	0.200
**Ex-smoker (vs current smoker)**	1.16	0.80	1.67	0.429
**Never smoked (vs current smoker)**	1.23	0.87	1.73	0.234
**Post-secondary education**	0.73	0.51	1.03	0.075
**Employed (vs unemployed)**	1.41	0.50	3.94	0.464
**Other employed status (vs unemployed)**	0.97	0.94	1.00	0.513
**BMI**	0.47	0.38	0.58	0.028
**Dispersion parameter estimate (θ)**	1.16	1.07	1.27	0.000

Logistic regression predicting probability of no disability days (structural zeros)

	OR	95% CI		p-value
**SBT score**	0.81	0.75	0.87	0.000
**PRS risk medium-low (vs low)**	0.98	0.69	1.39	0.921
**PRS risk medium-high (vs low)**	1.14	0.80	1.61	0.471
**PRS risk high (vs low)**	1.06	0.77	1.46	0.711
**Sex (male)**	1.40	1.10	1.79	0.007
**Ex-smoker (vs current smoker)**	1.03	0.75	1.43	0.835
**Never smoked (vs current smoker)**	1.28	0.95	1.74	0.109
**Post-secondary education**	0.78	0.55	1.11	0.165
**Employed (vs unemployed)**	0.71	0.38	1.32	0.278
**Other employed status (vs unemployed)**	1.29	0.55	3.02	0.563
**BMI**	0.96	0.93	0.98	0.001

Methods S1. The original English version of the STarT Back Tool (SBT) questionnaire.

The Keele STarT Back Screening Tool

Patient name: _______________________________ Date: _____________

Thinking about the **last 2 weeks** tick your response to the following questions:

		Disagree<br>0	Agree<br>1
1	My back pain has **spread down my leg(s)** at some time in the last 2 weeks	☐	☐
2	I have had pain in the **shoulder** or **neck** at some time in the last 2 weeks	☐	☐
3	I have only **walked short distances** because of my back pain	☐	☐
4	In the last 2 weeks, I have **dressed more slowly** than usual because of back pain	☐	☐
5	It's not really safe for a person with a condition like mine to be physically active	☐	☐
6	**Worrying thoughts** have been going through my mind a lot of the time	☐	☐
7	I feel that **my back pain is terrible** and **it's never going to get any better**	☐	☐
8	In general I have **not enjoyed** all the things I used to enjoy	☐	☐

9. Overall, how **bothersome** has your back pain been in the **last 2 weeks**?

Not at all	Slightly	Moderately	Very much	Extremely
☐<br>0	☐<br>0	☐<br>0	☐<br>1	☐<br>1

Total score (all 9): __________________ **Sub Score (Q5-9):**_____________

© Keele University 01/08/07
Funded by Arthritis Research UK

Methods S2. Finnish version of SBT.

Keele STarT selkäkysely

Tutkittavan nimi: ______________________________ Päiväys: _____________

Ajattele **viimeksi kulunutta 2 viikkoa** vastatessasi seuraaviin kysymyksiin:

		Eri mieltä 0	Samaa mieltä 1
1	Selkäkipuni on **säteillyt alaraajaani (-raajoihini)** jossakin vaiheessa viimeksi kuluneen 2 viikon aikana	☐	☐
2	Minulla on ollut **niska-** tai **hartiakipua** jossakin vaiheessa viimeksi kuluneen 2 viikon aikana	☐	☐
3	Olen **kävellyt ainoastaan lyhyitä matkoja** selkäkipuni vuoksi	☐	☐
4	Viimeksi kuluneen 2 viikon aikana **pukeutumiseni on ollut tavallista hitaampaa** selkäkivun vuoksi	☐	☐
5	Tällaisessa kunnossa olevan henkilön ei ole oikeastaan turvallista olla fyysisesti aktiivinen (harrastaa liikuntaa, työskennellä)	☐	☐
6	Olen ollut usein **huolestunut tilanteestani**	☐	☐
7	Minusta tuntuu, että **selkäkipuni on erittäin vaikea** eikä **se tule koskaan paremmaksi**	☐	☐
8	Yleisesti ottaen **en ole nauttinut** kaikista niistä asioista, joista ennen nautin	☐	☐

9. Kuinka **haittaavaa** selkäkipusi on ollut **viimeksi kuluneen 2 viikon** aikana?

Ei lainkaan	Hieman	Kohtalaisesti	Paljon	Erittäin paljon
☐	☐	☐	☐	☐
0	0	0	1	1

Kokonaispisteet (kaikki 9): ___________ Osapisteet (kysymykset 5-9):___________

© Keele University 01/08/07
Funded by Arthritis Research UK
